# Chitosan and Lecithin Ameliorate Osteoarthritis Symptoms Induced by Monoiodoacetate in a Rat Model

**DOI:** 10.3390/molecules25235738

**Published:** 2020-12-04

**Authors:** Reham Z. Hamza, Fawziah A. Al-Salmi, Nahla S. El-Shenawy

**Affiliations:** 1 Department of Biology, College of Sciences, Taif University, P.O. Box 11099, Taif 21944, Saudi Arabia; 2 Department of Zoology, Faculty of Science, Zagazig University, Zagazig 44519, Egypt; 3 Department of Zoology, Faculty of Science, Suez Canal University, Ismailia 41522, Egypt

**Keywords:** chitosan, lecithin, osteoarthritis, mitochondria, pathophysiology, rats

## Abstract

The present work aimed to assess the chondroprotective influence of chitosan and lecithin in a monoiodoacetate (MIA)-induced experimental osteoarthritis (OA) model. Forty male rats weighing 180–200 g were randomly distributed among the following five experimental groups (eight per group): control, MIA-induced OA, MIA-induced OA + chitosan, MIA-induced OA + lecithin, and MIA-induced OA + chitosan + lecithin. The levels of TNF-α, IL6, RF, ROS, and CRP, as well as mitochondrial markers such as mitochondrial swelling, cytochrome C oxidase (complex IV), MMP, and serum oxidative/antioxidant status (MDA level) (MPO and XO activities) were elevated in MIA-induced OA. Also, SDH (complex II) activity in addition to the levels of ATP, glutathione (GSH), and thiol was markedly diminished in the MIA-induced OA group compared to in control rats. These findings show that mitochondrial function is associated with OA pathophysiology and suggest that chitosan and lecithin could be promising potential ameliorative agents in OA animal models. Lecithin was more effective than chitosan in ameliorating all of the abovementioned parameters.

## 1. Introduction

Osteoarthritis (OA) is a severe debilitating and painful condition characterized by the gradual reduction of the articular cartilages, joints’ swelling, and then finally bone cysts [1]. There is no recent treatment for OA, and accessible therapy methods and analgesics for pain are currently prescribed to ameliorate pain [2]. Osteoarthritis pain constitutes a serious medical issue, however, OA animal models are being used excessively to elevate our awarness of OA-painful processes and to manifest the criteria of the new treatments.

Many patients are affected by OA, but currently, no remedies have been progressed to effectively cure OA disease and the current drugs only mititgate the symptoms of OA [3]. So, the only remedy available to OA chronic patients who reaching the last and final phases of OA is the replacement of joints, thus highlighting the urgent need to develop efficient and operative anti-OA treatment. One of the undetermined factors in OA development is the actual role of the inflammatory response and oxidative injury to the initiation and OA development.

During OA progress, a lot of inflammatory cytokines and prostaglandin E_2_, are increased in the joint bodies of OA patients and animals with OA-like diseases [4].

Additionally, inflammatory reactions such as aging along with other treatment interactions, have been demonstrated to afford oxidative injury by excessive production and triggering of H_2_O_2_ (Hydrogen peroxide), reactive oxygen species (ROS), superoxide anions and nitric oxide (NO), as well as by reducing the activity of the antioxidant enzymes [5]. However, OA has been considered previously to be a degenerative diseases rather than being an inflammatory diseases of the joints. Recently, researches have shown strong correlations between the pathogenesis of OA, the inflammatory actions, and oxidative injury [5,6,7]. Osteoarthritis is related to the elevated mortality of cartilage and chondrocytes.

Mitochondrial dysfunction can impact several procedures combined in the cartilage degradation and oxidative injury, increased cytokines, and the calcification of cartilage, as well as biosynthesis of the chondrocyte improperly [8].

Morphologically, multiple cristae compartmentalize the inner mitochondrial membrane, increasing the surface area, and maximizing the ability to generate ATP [8]. The inner membrane protein complexes are NADH dehydrogenase (complex I), succinate dehydrogenase (complex II), cytochrome c reductase (complex III), and cytochrome c oxidase (complex IV), and these transmit and discharge energy incrementally from the donated electrons used to pump protons (H^+^) into the intermembrane space. Altering the mitochondrial content can affect mitochondrial potential [9].

Chemical induced OA models allow us to study the lesions of OA at different stages. The monoiodoacetate (MIA) model which induced OA is the most common model used to evaluate the efficacy of the pharmacological agents in the treatment of the inflammation and severe pain. OA model can produce mimic quick pain-phenotypes that can be assorted by modifying the MIA dose [10,11]. The intra-articular injection of MIA in rodents induces OA lesions and can be examined. Monoiodoacetate is an inhibitor of glyceraldehyde-3-phosphatase that deactivates the cellular glycolysis and eventually leads to the cellular death [12]. In addition, MIA induces severe apoptosis of the chondrocytes, resulting in cartilage’s degradation and presence of bone osteophytes subsequently [13].

Perhaps the most prevalent polymer discovered in livestock, chitin can be hydrolyzed to produce chitosan, a material with very distinct characteristics, by a powerful alkali. These unique features of chitosan allow it to be used in different fields and medical applications [14]. Chitosan appears to promote tissue regeneration by allowing blood coagulation and enabling an endothelial layer to be attached to DeBakey-knitted grafts [15]. Additionally, chitosan has been shown to enable fast and scar-free healing in different animal species, which may be associated with enhanced cell membrane permeability and depends on administering the correct size of chitosan particles [16].

Lecithin is important for the cells of the body and it is prescribed to treat memory-related, gallbladder, liver, and skin diseases. Lecithin is also used to prevent the separation of certain components or as a food additive [17]. Few studies have explored the different biological activities of lecithin, but it is known that soy-derived lecithin has a considerable effect on improving liver function [18]. Since, the antioxidant activity of lecithin has been well-established [18], studying its impact on osteoarthritis could be helpful. Therefore, in this study, the effects of chitosan and lecithin on OA symptoms that were induced by monoiodoacetate (MIA) were investigated in details at the level of mitochondrial dysfunction, oxidative injury and TEM sections.

## 2. Materials and Methods

### 2.1. Chemicals

Chitosan and monoiodoacetate were purchased from Sigma, St. Louis, MO, USA. Lecithin was received from Doppelherz, Flensburg, Germany.

### 2.2. Induction of Osteoarthritis

Monoiodoacetate soln (MIA) in (sterile 0.9% sodium chloride) physiological saline at the recommended dose was freshly prepared. At the moment the animals were under light anesthesia, the MIA injection was administrated in a location around the knee joints.

### 2.3. Animals and Experimental Protocol

Forty male rats were divided into the following five groups (eight Rats /each): negative control (saline), MIA-induced OA (positive control), MIA-induced OA + chitosan, MIA-induced OA + lecithin, and MIA-induced OA + chitosan + lecithin. The control animals were administrated with physiological saline soln. (1 mL) as a vehicle. The positive (+ve) osteoarthritis (OA) control group was exposed to a single injection of 1 mg MIA in 30 µL of sterile physiological saline at the knee joints [19] and saline solution (1 mL) as vehicle, by using a sterile and sharp needle (i.p.) under light anesthesia by using sodium thiopental (40 mg/kg). No analgesic or anti-inflammatory drugs were given, for either the positive or negative control groups after MIA- injection or during the whole experimental period, in order to avoid interference with the obtained results. Ethics approval: All the animal experiments were approved by Zagazig University ethical committee under the approval number: Zu-IACUC/1/F/130/2019. No human experiments in the current study.

**Figure 1:**
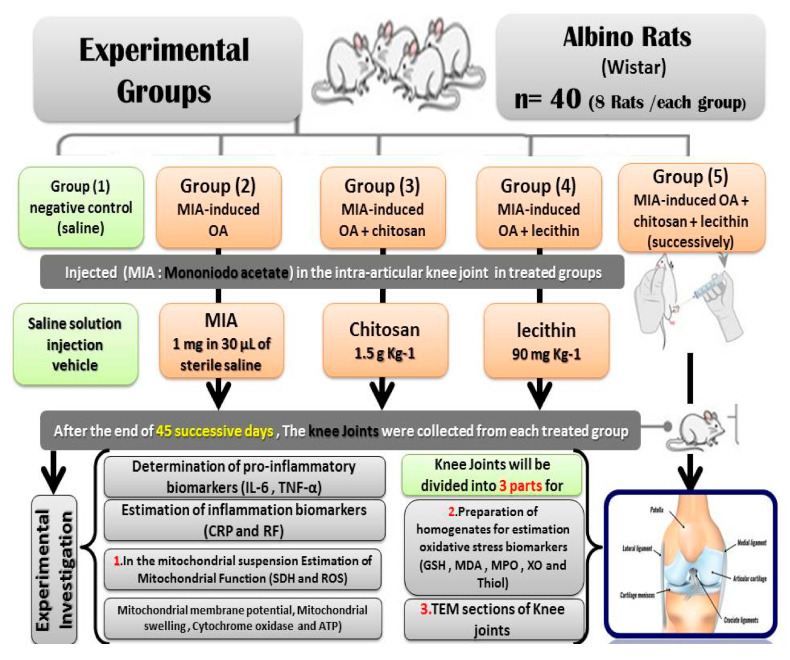
Experimental protocol.

The OA + chitosan rats were injected with MIA as previously mentioned, before being treated orally with chitosan at 1.5 g/Kg [20]. The OA + lecithin rats were injected with MIA and then treated orally with lecithin at a dose of 90 mg/Kg [21]. The final treated group (OA + chitosan + lecithin) was injected with MIA and then treated with both chitosan and lecithin, each at the doses described previously. The experimental period continued for 45 successive days. After the end of the experiment, All the rats were sacrificed under light anesthesia with ketamine/xylazine. Efforts were made to reduce the stress and pain of the rats; then, the knee joints (from the two knees of the rat) were divided into three parts. The first part was used for TEM sections, the second part for preparation of a mitochondrial suspension for measuring the bio-functional parameters of the mitochondria, and the third part was used for estimation of oxidative stress enzymatic and non-enzymatic parameters, as shown in Figure 1, which clarifies the experimental protocol.

Blood samples were collected to determine serum pro-inflammatory markers. Levels of (TNF-α) tumor necrosis factor-alpha and (IL-6) interleukin-6 were assessed by using Kits No. R6365 and RB1829, by using ELISA kits (BIOTANG INC, Cat. Lexington, MA, USA).

### 2.4. Estimation of Inflammation Biomarkers

The blood samples were also used to determine C-reactive protein (CRP) content via the method outlined in Wener et al. [22] using Enzyme-linked Immunosorbent Assay (ELISA) kits (SEA821). The detection of rheumatoid factor (RF) was depend on the capability of the sera of rheumatoid arthritis to be agglutinated [23] and was determined by using immunodiagnostic ELISA kits, Cortez Diagnostics, Calabasas, CA, USA).

### 2.5. Preparation of Mitochondrial Suspension from the Knee Joint for Evaluation of Mitochondrial Function

Firstly, rats were anesthetized using ketamine. The knee joint was isolated and homogenized in buffer solution, A 100 mL of mitochondrial isolation buffer was prepared by addition of 10 mL of 0.1 M Tris-4-morpholinepropanesulfonic acid and 1 mL of 0.1 M ethylene bis (oxyethylen enitrilo) tetraacetic acid to 20 mL of sucrose, adjusting the pH to 7.4, and bringing the volume to 100 mL with dist. H_2_O. Protease inhibitor was added in a 1:100 (volume) ratio to mitochondria isolation buffer. The homogenates were transferred into centrifuge tubes and were centrifuged at 1000× *g* for 20 min and at 48 °C, to pellet the nuclei and debris. The obtained supernatant was transferred to Eppendorf tubes and centrifuged in the Eppendorf Centrifuge for 10 min at 48 °C. The obtained supernatants were discarded; the pellets were resuspended in 100 mL of specialized ice-cold mitochondrial isolation buffer. The suspension was centrifuged to remove large particles and debris. The supernatant was discarded, and pellets were resuspended in 1 mL of mitochondria isolation buffer.

### 2.6. Estimation of Succinate Dehydrogenase (SDH, Complex II) Activity

The metabolic viability assay was assessed by using tetrazolium salts such as MTT (3-(4,5-Dimethylthiazol 2-yl)-2,5-diphenyltetrazolium bromide) were used to evaluate and measure the mitochondrial metabolic rate. Mitochondrial SDH activities were assessed by using the molecular probe MTT. The knee joints’ mitochondrial suspension was incubated with an MTT probe for about 1/2 h at 30 °C. Then, the formazan crystals were dissolved in 100 μL of dimethyl/sulfoxide. The amounts of formazan were directly proportional to the viable cells’ number. The final absorbance was estimated by a Microplate reader at absorbance of 570 nm (Tecan, Männedorf, Austria) [24].

### 2.7. Mitochondrial ROS Assays

The generation of mitochondrial ROS was estimated by using a dichlorodihydrofluorescein diacetate (DCFH-DA) probe. The knee joints’ mitochondrial suspension was incubated with (10 μM) DCFH-DA probe for about 0.5 h at 30 °C. The fluorescence intensity of dichlorofluorescein (DCF) was estimated by the Shimadzu RF-5000 U. fluorescence spectrophotometer (λ_ex_ = 488 nm, and λ_em_ = 527 nm). This technique depends on the use of 2,7-dichlorofluorescein diacetate, which enters the cell passively where it interacts with ROS to form the highly stable fluorescent compound dichlorofluorescein (DCF) which was measured and thus give an indication for ROS content. An increment in intensity of DCF indicated an increment in ROS generation due to exposure to severe osteoarthritis inflammation [25].

### 2.8. Mitochondrial Membrane Potential (MMP, ΔΨm) Assays

The mitochondria in all treated groups, were isolated from the knee joints. The generation of ROS from the mitochondria was assessed by using a rhodamine 123 (Rh123) probe for about thirty min at 30 °C. At the final step, the MMP collapse was estimated by using (Shimadzu RF-5000 U fluorescence spectrophotometer, Piscataway, NJ, USA (λ_ex_ = 490 nm and λ_em_ = 535 nm). An increment in the fluorescence intensity of (DCF) indicated an increment in the MMP collapse [26].

### 2.9. Evaluation of Mitochondrial Swelling

Briefly, mitochondrial swelling in sizes of 10 and 100 nm was measured in isolated mitochondria by using an ELISA (microplate reader) (Tecan, Rainbow Thermo) at 540 nm. Rat knee joints mitochondria were isolated as described previously. Mitochondrial swelling was measured with ELISA (microplate reader) that measured absorbance at 540 nm in a medium containing mitochondria (0.25 mg/mL) and (in mM) 125 KCl, 20 HEPES, 2 KH_2_PO_4_, 0.025 EGTA, 4 MgCl_2_, 0.2 ATP, 5 malate, and 5 glutamate, pH 7.08 (titrated with KOH) at 37 °C. A decline in absorbance indicated an elevation in the mitochondrial swelling [26].

### 2.10. Assay of Cytochrome-C Oxidase Release

Estimation of cytochrome c release in mitochondrial suspension, which indicates the mitochondrial volume, was estimated by using Quantikine Rat/Mouse cytochrome c immunoassay kits (Minneapolis, MN, USA).

### 2.11. Assays of ATP Content 

The content of ATP was detected by “luciferase enzyme” in mitochondrial lysate, and intensity was evaluated using a Sirius tube luminometer (Berthold Detection System, Bad Wildbad, Germany). ATP content was expressed as nmol mg^−1^ protein [27].

### 2.12. Preparation of the Knee Joint Tissue Homogenates

A small portion of the knee joint tissues was used for estimating antioxidant biomarkers. Knee joint tissues were washed three times with saline for the removal of blood. Tissues were immersed with a sodium phosphate buffer (pH 7.4) in an ice medium and centrifuged at 5000 rpm for 1–2 h. The obtained supernatant was stored at −80 °C for further use.

### 2.13. Glutathione (GSH) Content Assays

Glutathione content was measured in the knee joint homogenates. The knee joint homogenates were added to phosphate buffers and DTNB (pH 7.4). The obtained yellow color was read spectrophotometrically by using a spectrophotometer (UV-1601 PC, Shimadzu, Japan) at 412 nm. Glutathione content was expressed as μg mg^−1^ protein [28].

### 2.14. Lipid Peroxidation (MDA) Assays

The malondialdehyde (MDA) was estimated in the knee joint homogenates to measure MDA. MDA level was estimated at 532 nm by using an ELISA (microplate reader) (Tecan, Austria). Additionally, MDA level was expressed as μg mg^−1^ protein and an elevation in MDA indicated an increment in LPO [29].

### 2.15. Myeloperoxidase (MPO) and Xanthine Oxidase (XO) Activities

Myeloperoxidase and XO were evaluated in the knee joint homogenates spectrophotometrically according to the methodologies outlined by Suzuki et al. [30] and Litwack et al. [31], respectively.

### 2.16. Estimation of Total Thiol Levels

Thiol levels were determined in the knee joint homogenates by using the methodology detailed by Hu [32] and were presented as mmol g^−1^ tissues.

### 2.17. TEM Examinations (Transmission Electron Microscope)

Knee joint portions were dissected and TEM sections were examined by fixing the samples in 2.5% glutaraldehyde and then, embedding them in resins [33]. Specimen block was cut into ultrathin sections (0.05 mm), and then the sections were stained with uranyl acetate for 1 h and then, stained with lead citrate for about 15 min. Knee joint sections were examined under a transmission electron microscope unit (JEOL, Akishima, Tokyo, Japan, 12,000× magnification) at Mansoura University, Faculty of Agriculture, Mansoura, Egypt.

### 2.18. Statistical Analysis

Statistical analysis was performed by using SPSS software version 27 [34] and Open Epi version 2.3.1 [35]. The graphical and tabular presentation was done. Data were summarized as the mean and standard error. Shapiro-Wilk test was used to determine the distribution characteristics of variables and variance homogeneity. One way ANOVA and post hoc power were used to analyze data. A *p*-value of ˂0.05 was accepted as statistically significant [36].

## 3. Results

### 3.1. IL-6, TNF-α, and CRP Levels in the Serum

**Figure 2:**
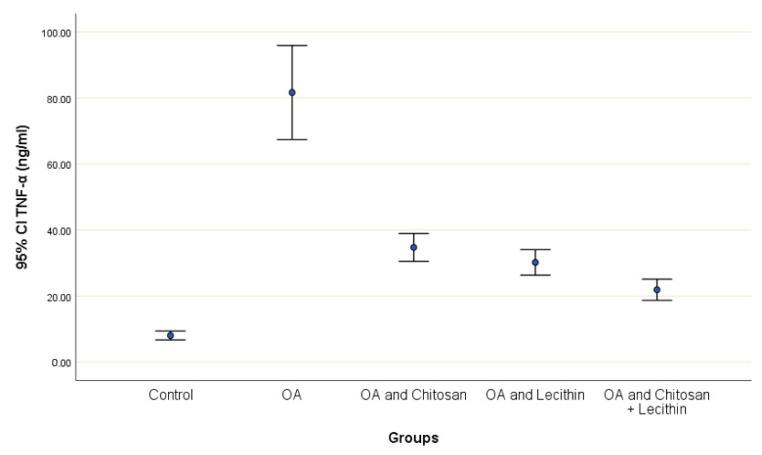
The efficacy of chitosan and lecithin against tumor necrosis factor-alpha (TNF-α) in (OA) model induced by monoiodoacetate (MIA). The data presented as mean ± S. E.

**Figure 3:**
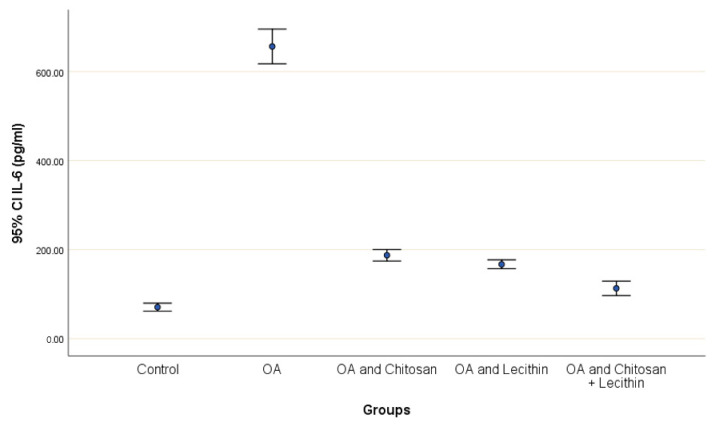
The efficacy of chitosan and lecithin against interleukin-6 (IL-6) in (OA) model that was induced by monoiodoacetate (MIA). The data presented as mean ± S. E.

TNF-α and IL-6 levels were revealed in serum at the end of the experiment after injection with MIA (Figure 2 and Figure 3). The levels of TNF-α and IL-6 were elevated markedly in the control OA treated group, as compared to those in the negative control groups. Chitosan and lecithin both suppressed TNF-α levels, while the combination of both decreased the TNF-α level. Chitosan, lecithin, and the two in combination decreased IL-6 levels as compared to the positive OA control treated group.

**Figure 4:**
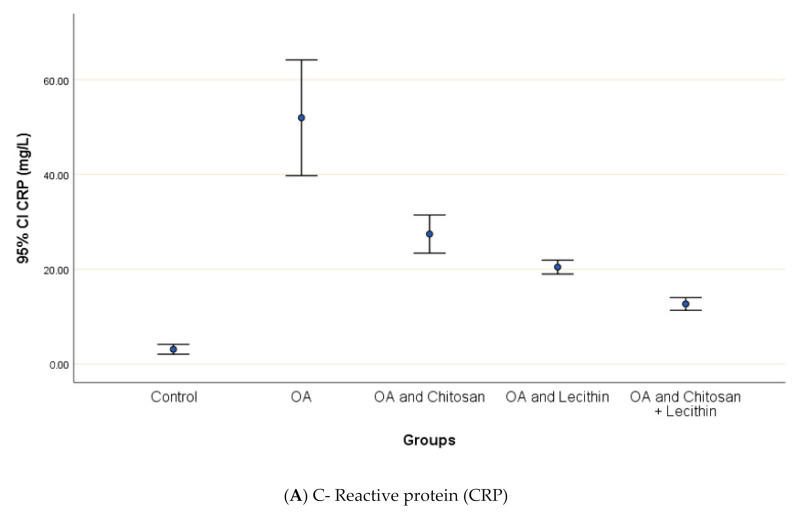

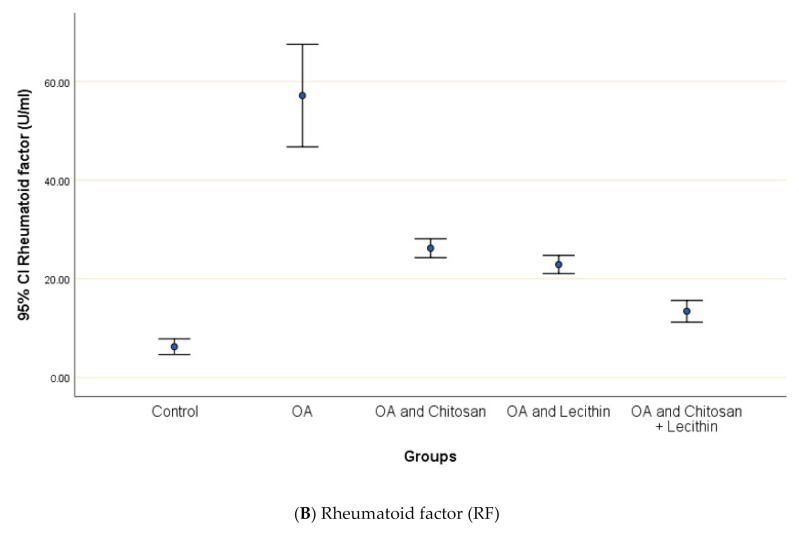
The efficacy of chitosan and lecithin against: (**A**) C-reactive protein (CRP) and (**B**) rheumatoid factor (RF) of (OA) model that was induced by monoiodoacetate (MIA). The data presented as mean ± S. E.

CRP serum level in control group was 3.12 ± 0.5 mg/L, with a marked significant difference and OA-rats (Figure 4). Administering chitosan to OA-animals reduced CRP levels. In comparison to the positive control (MIA-induced OA) animals, rats treated with lecithin showed an average reduction in CRP level on day 45. Significantly, CRP levels were more declined in rats treated with chitosan and lecithin compinations as compared to the declines observed under either treatment in isolation.

### 3.2. SDH, ROS, and MMP

The levels of SDH, ROS and MMP were significantly elevated in OA group, as compared to those in negative control group (Figure 4). Treatment of the rats with chitosan, lecithin, or both significantly lowered the RF, as compared to the positive control group (MIA-induced OA).

**Figure 5:**
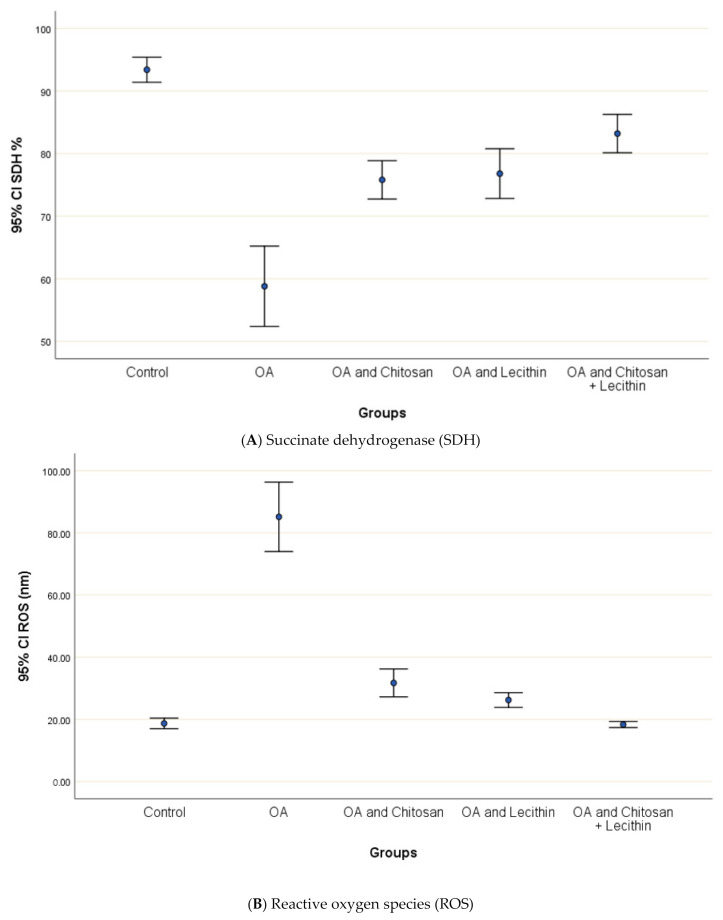
The efficacy of chitosan and lecithin against: (**A**) succinate dehydrogenase (SDH) and (**B**) reactive oxygen species (ROS) in (OA) model induced by monoiodoacetate (MIA) in male rats. Where Fluorescence intensity (Relative wave length and content for DCF) (Indication for ROS content). The data presented as mean ± S. E.

The baseline ROS value for normal control rats was 18.7 ± 1.1 nm (Figure 5). The (MIA-induced OA) arthritic rats showed higher mitochondrial ROS contents than the negative control rats, and ROS generation was decreased significantly in chitosan-treated rats. Greater reductions in ROS content were observed in the lecithin alone and lecithin + chitosan treatments, as these treatments reduced ROS contents greatly, respectively.

**Figure 6:**
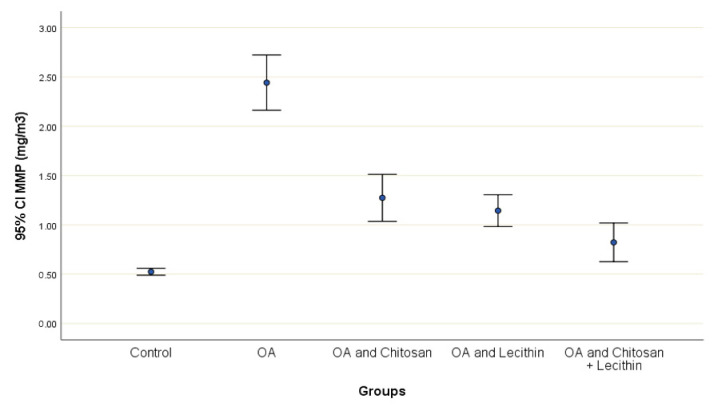
The efficacy of chitosan and lecithin against mitochondrial membrane potential (MMP) in (OA) model induced by monoiodoacetate (MIA) in male rats. The data presented as mean ± S. E.

The effects of chitosan and/or lecithin on MMP (Δψm) are clarified in Figure 6. The MIA-induced OA-model presented significantly elevated MMP as compared with the healthy control animals, and this variable was notably decreased by the chitosan + lecithin, chitosan, and lecithin treatments, in descending order. The current results suggested that chitosan and lecithin administered in combination remarkably reduced the MMP collapse in OA group.

### 3.3. Cytochrome-C Release, Mitochondrial Swelling and ATP content

**Figure 7:**
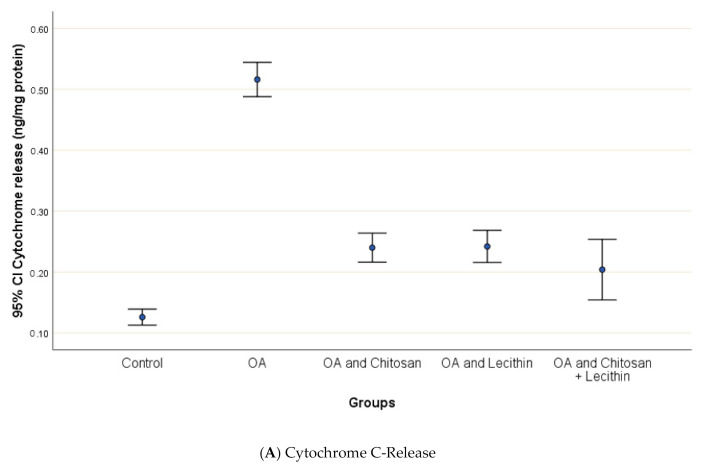

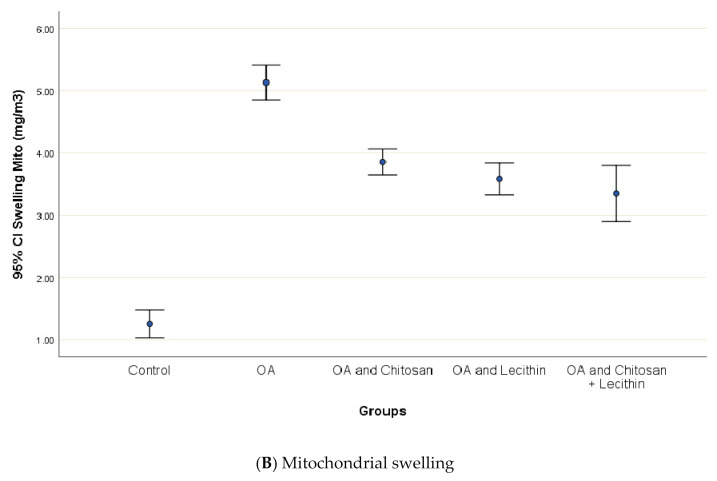
The efficacy of chitosan and lecithin against: (**A**) release of Cytochrome-C and (**B**) the mitochondrial swelling in (OA) model induced by monoiodoacetate (MIA) in male rats. The data presented as mean ± S. E.

Osteoarthritis accelerated the mitochondrial swelling, as demonstrated in Figure 7, as it was significantly increased in MIA-induced OA as compared to the control group. The mitochondrial swelling was diminished significantly under the chitosan, lecithin, and the combination treatment, respectively.

**Figure 8:**
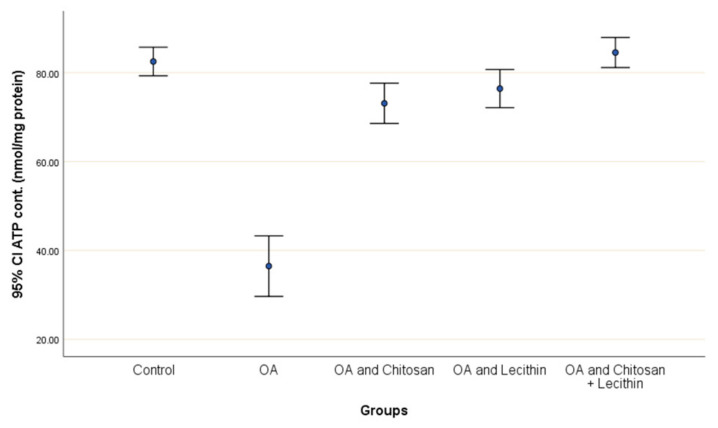
The efficacy of chitosan and lecithin against ATP contents in the (OA) model induced by monoiodoacetate (MIA) in male rats. The data given as mean ± S. E.

Positive control rats had a lower ATP content (55.8%) compared to negative control rats (Figure 8). The treatment with chitosan elevated the ATP content significantly as compared to OA rats, and the lecithin treatment elevated ATP content significantly. The chitosan + lecithin combination treatment lead to a significantly greater ATP content as compared to OA group than either treatment administered in isolation.

### 3.4. Mitochondrial Oxidative Stress and Antioxidant Elevation

The MDA levels in the OA treated rats were more higher than that of the negative control rats (Table 1). This was accompanied with a significant decrease at the GSH contents. In contrast, treatment with chitosan, lecithin, or both produced a significant decreasing effect on MDA levels as compared to positive control male rats. Elevations of GSH were also observed under the chitosan, lecithin, and chitosan + lecithin treatments, respectively, in comparison to the positive control (MIA-induced OA) group (Table 1).

Myeloperoxidase and XO levels increased significantly in positive control group as compared to control rats, respectively (Table 1). Male rats treated with chitosan exhibited significantly decreased MPO and XO activities as compared to the MIA-induced OA group. Treatment with lecithin also produced a significant decrease in MPO and XO levels as compared to OA control rats. The chitosan + lecithin treatment declined the MPO and XO activities as compared to OA control male rats, respectively.

Thiol levels were significantly decreased in OA treated group as compared to control male rats (Table 1). Increasing the thiol levels were obtained in a group treated with either chitosan, lecithin/or both.

### 3.5. Electron Microscopy Evaluations

**Figure 9:**
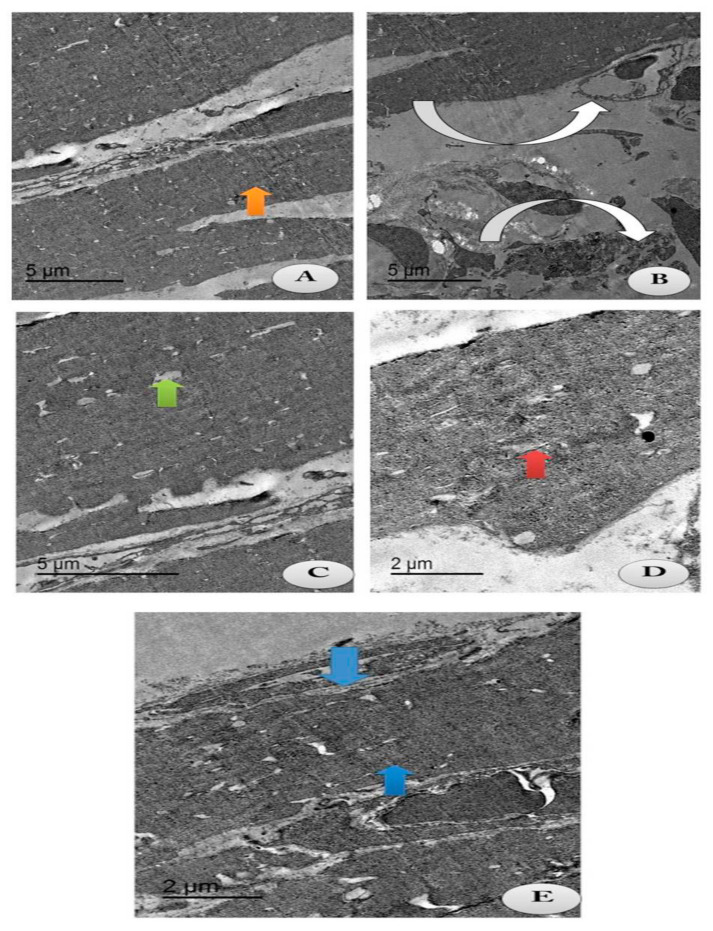
TEM section of the changes in the articular cartilage of the knee joints post-treatment with either chitosan and/or lecithin or combined after OA induction. Images showing collagen fibers of the following treated groups. (**A**) Control group: showing intact cartilage fibers (Orange arrow) with appearance of intact articular septa and intact chondrocyte (Green arrow) (Scale bar = 5 µm). (**B**) OA group (+ ve control group): showing atrophy of cartilage fibers (Inverted white arrows) with severe distortion of the articular septa (Whitehead arrow) and reduction in chondrocytes (Green arrow) (Scale bar = 5 µm). (**C**) OA group + Chitosan: showing some reduced chondrocyte (Green arrow) with most intact cartilage fibrils with the irregular articular septum (Inverted white arrow). (Scale bar = 5 µm). (**D**) OA group + Lecithin: showing moderate chondrocyte size (Red arrow) with intact cartilage fibrils (Inverted white arrow) and mild wide articular septum (Scale bar = 2 µm). (**E**) OA group + Chitosan +Lecithin: showing mostly high restoration of cartilage fibrils (Blue arrow) with mostly normal chondrocytes’ size (Green arrow) with appearance of intact and compact cartilage fibers and high restoration of the intra-articular septum (Scale bar = 2 µm). Table 2 clarifies the morphometric changes in all the treated groups and the rate of change in each treated group.

The current study aimed to identify whether chitosan and/or lecithin improved the degeneration of the articular cartilage on OA-treated rats (Figure 9). An intact cell structure was clarified in the control group, with appearance of intact fibers of the cartilage and normal articular septum (Figure 9A). Cartilage atrophy of OA with severe distortion of the articular septum was appeared in the cytoplasm in the positive OA control group (Figure 9B). Following treatment with chitosan, some reductions in the chondrocytes’ size with mostly appearance of intact cartilage fibrils and an irregular articular septum were observed, as compared to the OA-treated animals (Figure 9C). These results indicated that chitosan alleviated the degeneration of chondrocytes caused by OA. The same observations were noticed in OA-animals treated with lecithin, as moderate chondrocytes’ size with appearance of intact cartilage fibrils and a mild wide articular septum were noted (Figure 9D). The osteoarthritic group treated with both chitosan and lecithin showed restoration of the majority of cartilage fibers with majority appearance of normal chondrocytes’ size with presence of intact cartilage fibrils and mostly restoration of the inner articular septum (Figure 9E). TEM scoring and morphometric changes in all fields were recorded in (Table 2).

## 4. Discussion

Arthritis causes disability due to pain and severe inflammation in joints. Osteoarthritis is one of the many forms of arthritis, the prevalence of which increases with age. It occurs in various joints, including the knee, which is prevalent. OA is ranked as the 11th highest contributor to global disability in the world [37].

The MIA model can induce OA lesions rapidly in the rats, comparable to the progression present in the human OA-disease. The OA model can be used to evaluate chondral lesions or to study potential OA drugs [12].

Cartilage, which is attached to the bones’ ends in joints, is lack of blood vessels. Instead, it bathes in a liquid that performs as a lubricant. Within the thin tissue, macrophages found that have two purposes: Firstly, to protect and nourish the cartilages, and to preserve the knee joints against any infections. Joints’ inflammation can be developed and in case of chronic inflammation; macrophages start in producing molecules that degenerate the cartilages [38].

Osteoarthritis is distinguished by the degeneration of the articular cartilages, severe inflammation, mortality of the synovial fluids, joints and chondrocytes [39]. Several factors have been recommended to cause OA, including chronic inflammation and oxidative stress [40]. The excessive triggering of cytokines increases reactive oxygen species (ROS) levels by activating neutrophils and macrophages, causing them to become joint-damaging [41]. Thus, the excessive accumulation of reactive oxygen species occurred in the MIA-induced OA in male rats.

There is no treatment for osteoarthritis to date; however, many animal studies have provided rationale for the use of antioxidant supplements for osteoarthritis management [42]. Therefore, in the current study, we assessed the reality of the benefits and synergistic effect of using both chitosan and lecithin, as well as their combination, as potent antioxidant supplements for the management of knee osteoarthritis.

In the present study, there was significant improvement in the knee joints of groups treated with either chitosan and/or lecithin, while great improvement was recorded in the osteoarthritic group which treated with a combination of chitosan and lecithin, as well as a marked depletion in markers of inflammation and oxidative stress.

Inflammatory cytokines are multi-effective proteins that have major roles in the cellular conjugation and communication. TNF-α is an major cytokine as it controls a lot of pathological events, including cellular swelling, inflammation and even apoptosis [39]. It is reported that there is growing evidence that CRP has a vital function in the inflammatory process and that CRP levels rise in circulation [43].

IL-6 and TNF-α play major roles in modifying OA inflammatory series, they can give a real picture on the severity of inflammation [43] and are known to induce a lot of catabolic events as they enhance MMP [44]. TNF-α down-regulates the synthesis of the components in the cellular matrix by suppressing the chondrocyte anabolic activities and by reducing the production of collagen type II [44].

The production of IL-6 by chondrocytes is small under normal physiological circumstances. However, the precise cause of IL-6 activity in OA may be due to the number of cytokines and growth factors present during OA, such as IL-1β, TGF-β, and prostaglandin, that stimulate its production. Moreover, increased levels of serum IL-6 have been correlated with the severity of OA lesions [44] and these findings confirmed the obtained results by a high level of Il-6 in the OA positive control group and its reduction in OA after treatment.

There is appropriate evidences that cartilage is damaged during OA [45]. The major two cytokines that are responsible for inflammation (IL-6 and TNF-α) have been correlated to the progression of knee cartilage loss [46], which may be because IL-6 and TNF-α reflect the development of hyperplasia of the synovial fluids, which leading to OA- advancement and growth [46]. The data recorded in the present study is in complete agreement with Wojdasiewicz et al. [47], who demonstrated that TNF-α and cytokine inflammatory marker levels were elevated in OA joints and synovial fluid as compared to in healthy individuals.

The main cause of OA may be the excessive accumulation and generation of ROS, which causes increased MDA levels [48]. In the current study, LPO levels were elevated and indicated cellular membrane damage. Enzymatic antioxidant (SOD and CAT) levels were declined as these regulate O2^−^ and H_2_O_2_ levels. Moreover, the thiol and GSH endogenous antioxidants were declined in the MIA-elicited OA in male rats.

Additionally, it was demonstrated that RF is considered as an essential tool in the polyarthritis’ diagnosis because they make the identification of rheumatoid patients possible [49]. Rheumatoid factors also were used previously to predict the active action of TNF-α in the current findings, and there was a positive and direct relation was found between both of TNF-α and RF.

In the present study, there was a marked improvement in the knee joints of groups treated with either chitosan and/or lecithin, and the great improvement was proved and recorded in the osteoarthritic group treated with successive treatment combination of chitosan and lecithin as well as a marked depletion in markers of inflammation and oxidative stress.

The obtained findings are in agreement with those of Oprenyeszk et al. [50], who confirmed the anti-inflammatory and catabolic changes in the chondrocytes in the case of treatment of OA human chondrocytes with chitosan with alignate encapsulation, as chitosan capsulate reduced the production of inflammatory and catabolic mediators by OA chondrocytes and tended to stimulate the synthesis of cartilage matrix components. This reinforces our data. The novelty of our study is the use of both chitosan and lecithin, which we believe can empower the anti-inflammatory properties of chitosan.

Confirming the anti-inflammatory effects of chitosan, Wang et al. [51] reported that OA increased collagen II expressions and that the combination of chitosan and hyaluronic acid significantly decreased the classification and pathological scores to be close to the normal level.

One component of chitosan is β-d-glucosamine, which may help to reduce inflammation and pain, as well as normalize synovial fluid viscosity and promote repair of joint cartilage affected by OA. Glucosamine is a dietary supplement commonly used by OA patients and is recommended by physicians for its purported analgesic and chondroprotective effects [52]. Glucosamine is one of the most abundant monosaccharides in the human body and is present in the articular cartilage in high quantities [52].

A lecithin formulation with some standardized natural extracts had a distinguished and marked effect on reducing the knee OA symptoms (both pain and knee function), due to the anti-inflammatory activity showed by a reduction of inflammation markers (CRP) [53], similar to the results obtained in our current study for the OA group treated with Lecithin, with a greater reduction in inflammatory markers in the case of treatment with both chitosan and lecithin. These results are very interesting, novel and appealing, and so we can hypothesize that the decrease of pain and the improvement of the knee function in the case of treatment of OA animals with both Chitosan and lecithin demonstrates their synergistic effect in the alleviation of OA pain.

In the current study, lecithin was examined for its anti-inflammatory effects, as it may have pharmaceutical potential. Lecithin decreased the levels of TNF-α, IL6, CRP, RF, ROS, MMP, mitochondrial swelling, complex IV, and MDA, as well as the activities of MPO and XO, to a greater extent than chitosan in MIA-induced OA in rats, compared to positive control OA-group; however, these decreases were not significant. SDH activity (complex II) and the ATP levels, thiol and GSH were markedly elevated in OA group treated with lecithin, as compared to positive control OA group.

These findings suggest that lecithin may have anti-arthritic qualities. These observations may be due to lecithin containing many enzymatic and non-enzymatic antioxidants, thereby allowing it to insert itself into the cell membrane and maintain its integrity, such that it can react with free radicals by scavenging them and preventing LPO [54]. Moreover, the multiple therapeutic activities of lecithin are associated with its anti-inflammatory and antioxidant effects [55,56]. Hence, it can be concluded that lecithin possesses anti-inflammatory qualities.

Lecithin administration was effective in reducing the inflammatory process through inhibition of different molecules involved in inflammation, including TNF-α and IL-6, compared to OA control animals, as previously described in Ali et al. [57]. The elevation of MPO activity is a major effective diagnostic tool for oxidative injury biomarkers and inflammatory markers in OA and other immune diseases [58]. Naegelen et al. [59] demonstrated that the oxidative injury displays a vital and effective role in the triggering of cellular MPO. Myeloperoxidase triggers ROS (reactive oxygen species) and excessive free radicals depending on the accessibility of the substrates [60].

Lecithin is the precursor for choline and, so, it regulates cellular membrane permeability and keeps the fats circulating in the bloodstream in balance. Lecithin has been found to lower inflammatory biomarkers [61]. Moreover, curcumin and lecithin complexes have also been shown to enhance chondroprotective effects through different anti-inflammatory mechanisms [37].

During OA, the degradation of neutrophils results in the release and discharge of the cellular enzymes, which eventually leads to oxidative stress [44]. Thus, increased MPO in the cartilage, which has been connected with the development of this disease [45].

The mitochondrial membrane potential plays an essential role in the development of cartilage degradation in OA due to its capacity to cleave various extracellular matrix components [37,61]. Several studies have verified that the inhibition of MMP postpones the progression of in vitro and in vivo cartilage degradation [62] where fibrillar collagens are irreversibly damaged [63].

In the present study, OA control animals showed decreased enzyme mitochondrial activities of complexes II and IV, as well as reduced ΔΨm. Moreover, osteoarthritic rats displayed elevated mitochondrial mass. These findings could be the result of mechanisms to improve the oxidative phosphorylation yield per cell. Zorova et al. [64] reported that the greater the ΔΨm, the higher the internal mitochondrial membrane’s energy capacity and the greater the ATP synthesis.

In the current findings, in control and OA-treated groups, the vitality of mitochondria was assessed by analyzing the enzymes of the respiratory chains [65]. The mitochondrial dysfunction in the positive control (MIA-induced OA) rats was characterized by decreased it’s activity as compared to control rats, as also found in Blanco et al. [66]. Ensuring mitochondria respiratory chain (MRC) integrity is important for producing ATP and maintaining mitochondrial membrane potential [67]. Since mitochondrial depolarization responsible for the depletion of ATP, the ΔΨm in chondrocytes of OA rats were evaluated in the present work.

Mitochondrial membrane potential (ΔΨm) is an important factor that determines the mitochondrial viability and it is implicated in the removal of the mitochondrial degradation [68]. The mitochondrial collapsing is related with the mitochondrial swelling and the liberation of apoptotic factors as cytochrome-c [65]. All the obtained results are symmetrical with the findings obtained in the current study and previous studies that have observed more and severe apoptotic chondrocytes in OA treated group than in normal cartilages [64,65,66]. Nevertheless, the degradation of ΔΨm causes ATP depletion, and apoptosis involves caspase activation based on ATP.

Given that excessive ROS production can immediately trigger various pathologies, retaining excessively elevated mitochondrial ΔΨm is possibly detrimental to mitochondria and therefore to the cell [64].

Cytochrome-c which is released from the mitochondria to the cytosolic part represent the first step in the cellular apoptosis, as to liberate a caspase cascade that inter the cells to the cellular death cycle. It has been reported that the mitochondrial matrix swelling triggered due to any stimuli that may cause apoptosis causes the major cytochrome-c discharge [68], and this parallels the current findings that cytochrome c levels were significantly elevated in OA control group.

The current TEM structure of the OA group clarified a significant reduction in chondrocytes and cartilage fibril degeneration, as confirmed previously by Ilas et al. [69], who confirmed degeneration and lesions in knee joints by MRI, while treatment of the OA group with either chitosan and/or lecithin significantly improved the ultrastructural investigation of joints and restored, to a greater extent, the cartilage fibrils and enlarged the chondrocytes. These current results are in line with those of Oprenyeszk et al. [50], who found that the ultrastructure (TEM) of Chondrocytes cultivated with a chitosan capsulation led to an enlargement of the chondrocyte matrix and intact fibrils. This confirms our new finding, demonstrating that the combination of chitosan and lecithin can lead to a greater effect on the ultrastructure of the cartilage and improve the chondrocyte fibrils.

In conclusion, the modifications induced by OA inflammation represent the main imbalanced case that essentially contributes to the energy metabolism and the immune responses to the OA diseases. The mitochondrial membrane potential is well-known to provide an early marker of cellular apoptosis. chitosan and lecithin were shown to have a lot of therapeutic benefits, reducing oxidative stress and suppressing the inflammatory levels in OA group. These current findings suggested the potent therapeutic activities of chitosan and lecithin for the possible active treatment of most of the autoimmune diseases via the mitochondrial pathway, without eliciting any side effects associated with the standard therapies. Our data were proven by electron microscope examination.

## Figures and Tables

**Table 1:** The efficacy of chitosan and lecithin against oxidative and the antioxidant status in the osteoarthritis (OA) model that was induced by monoiodoacetate (MIA).

	GSH (µg/mg Protein)	MDA (µg/mg Protein)	MPO (nmol/min/g)	XO (U/g)	Thiol Level (mmol/g Tissue)
**Group 1**	0.79 ± 0.06	0.52 ± 0.02	22.82 ± 2.05	15.81 ± 1.04	16.26 ± 0.81
**Group 2**	0.40 ± 0.09 ^a^	1.37 ± 0.17 ^a^	33.59 ± 1.91 ^a^	31.49 ± 5.05 ^a^	8.79 ± 1.17 ^a^
**Group 3**	0.57 ± 0.04 ^b^	0.69 ± 0.05 ^b^	29.47 ± 2.44 ^b^	26.16 ± 2.94	12.71 ± 1.02 ^b^
**Group 4**	0.61 ± 0.03 ^b^	0.64 ± 0.06 ^b^	27.14 ± 4.62 ^b^	23.87 ± 3.26 ^b^	14.27 ± 0.90 ^b^
**Group 5**	0.73 ± 0.09 ^b,c^	0.53 ± 0.03 ^b,c^	23.76 ± 3.66 ^b^	19.03 ± 1.86 ^b,c^	15.58 ± 0.73 ^b,c^
**Post hoc power analysis**					
**Groups 1 versus 2**	100%	100%	100%	100%	100%
**Groups 1 versus 3**	100%	100%	100%	100%	100%
**Groups 1 versus 4**	100%	100%	77.12%	100%	99.94%
**Groups 1 versus 5**	41.85%	13.92%	10.47%	99.76%	50.49%
**Groups 2 versus 3**	99.98%	100%	98.76%	82.24%	100%
**Groups 2 versus 4**	100%	100%	98.3%	97.98%	100%
**Groups 2 versus 5**	100%	100%	100%	100%	100%
**Groups 3 versus 4**	71.57%	52.58%	29.13%	37.82%	95.22%
**Groups 3 versus 5**	99.93%	100%	98.4%	100%	100%
**Groups 4 versus 5**	97.93%	99.94%	44.18%	98.29%	94.68%

The data presented as mean ± S. E. ^a^ The differences are significant in comparison with the control group (*p* ≤ 0.05). ^b^ significance different as compared to the OA group. ^c^ Significance different as compared to OA with lecithin.

**Table 2:** TEM findings, Scoring and Morphometric changes in Knee joint tissues of different treated groups (In the examined fields of rats of each group).

Findings	Control Group	OA	OA and Chitosan	OA and Lecithin	OA and Chitosan + Lecithin
Intact compact cartilage fibers	++++	------	---+	---+	-+++
Atrophy of cartilage fibers	------	++++	---+	---+	------
Changes in joint structure following OA	------	++++	--++	---+	------
Reduced chondrocyte	------	++++	------	------	------
Cartilage degeneration	------	++++	---+	---+	------
Chondrocyte viability	++++	------	-+++	-+++	++++
Catabolic changes	------	-+++	---+	---+	------

------ Absence of the change in the animals of the studied group; ++++ A change was observed in 90% of the group; -+++ A change was observed in 80% of the group; --++ A change was observed in 50% of the group; ---+ A change was observed in 25% of the group.
